# Low positive affect display mediates the association between borderline personality disorder and negative evaluations at zero acquaintance

**DOI:** 10.1186/s40479-019-0103-6

**Published:** 2019-03-03

**Authors:** Johanna Hepp, Susanne Gebhardt, Pascal J. Kieslich, Lisa M. Störkel, Inga Niedtfeld

**Affiliations:** 1 Department of Psychosomatic Medicine, Central Institute of Mental Health, Medical Faculty Mannheim at Heidelberg University, J5, 68159 Mannheim, Germany; 2 Department of Psychology, School of Social Sciences, University of Mannheim, Mannheim, Germany

**Keywords:** Borderline personality disorder, Thin slices, Zero acquaintance, Positive affect, Affect expression, Facial affect, Gaze, Eye contact, Trustworthiness, Cooperation

## Abstract

**Background:**

Several recent studies have demonstrated that naïve raters tend to evaluate individuals with Borderline Personality Disorder (BPD) negatively at zero-acquaintance (i.e., in a ‘first impression’ type situation, where the rater has no knowledge of the individual and no prior interactions with them). Specifically, individuals with BPD were evaluated as less trustworthy, likeable, and cooperative than healthy participants (HCs). Based on previous impression formation studies, we hypothesized that the non-verbal cues positive affect display, negative affect display, and eye contact contribute to negative first impressions of those with BPD.

**Methods:**

To address this question, we recruited 101 participants that rated the degree of positive affect display, negative affect display, and eye contact in 52 videos of age-and gender-matched BPD and HC participants. We hypothesized that low positive affect display, high negative affect display, and eye contact would mediate the association between group (BPD vs. HC) and ratings of trustworthiness, likeability, and cooperativeness.

**Results:**

Ratings for positive affect display were significantly lower and those for negative affect display significantly higher for BPD versus HC targets, whereas eye contact did not differ significantly between groups. In multiple mediation models, positive affect display significantly mediated the association between group and trustworthiness/likeability, whereas negative affect display only mediated the association between group and likeability. None of the individual cues was a significant mediator of the association between group and cooperation.

**Conclusions:**

We emphasize therapeutic possibilities to improve positive affect display –and thus overall first impressions– to increase the chances of forming social bonds for BPD individuals.

## Background

Borderline Personality Disorder (BPD) is a serious mental illness that afflicts between 1 and 3% of the adult population and starts to manifest in late childhood or early adolescence [1, 2]. Individuals with BPD have intense and rapidly changing emotions, tend to show impulsive and self-harming behaviors, and suffer from interpersonal problems [1]. The present study focuses on factors that contribute to interpersonal problems in BPD, which represent a core symptom of the disorder and are among the slowest BPD symptoms to remit [3, 4]. Interpersonal dysfunction in BPD manifests in various ways, but prominent examples include small social networks [5], high levels of romantic relationship dysfunction [6], and extreme loneliness [7].

In light of the central role interpersonal problems play in BPD, it is not surprising that much research has been devoted to identifying factors that contribute to their manifestation. Central factors that have been identified include impairments in social cognition, deficits in cooperative behavior, and functional neuronal alterations (for an overview, see [8–10]). Beyond these factors pertaining to altered processes on the part of the BPD individual, recent studies have also suggested that naïve raters form negative first impressions of those with BPD. Specifically, there is evidence that naïve raters tend to view BPD individuals negatively, which could contribute to interpersonal problems by way of negative behavior towards the BPD individual.

Most studies that assess how BPD individuals are perceived by others have focused on populations of health professionals and their attitudes towards BPD patients. These studies show that health professionals tend to evaluate BPD patients more negatively than other patient groups on dimensions such as likeability (for a review, see [11]). Beyond these, there is also a small number of studies that assessed how BPD individuals are perceived at zero-acquaintance (i.e. in a ‘first impression’ type situation where the rater has no knowledge of the individual and no prior interactions with them), that is, when their diagnosis is not known. A series of studies by Daros and colleagues [12] showed that, based on photographs, BPD individuals were evaluated as more mentally and physically ill than healthy participants at zero-acquaintance. Additionally, BPD individuals were attributed more negative emotions and less happiness than healthy control individuals in this sample. Additional research on zero-acquaintance judgments of BPD individuals was conducted by Oltmanns and colleagues [13] as well as Friedman and colleagues [14], who collected large target samples of military personnel and over-sampled for personality disorder features. Short video sequences of targets talking about ‘things they enjoy doing’ were shown to student raters. Raters evaluated targets with high BPD features low on likeability, extraversion, agreeableness, openness, and conscientiousness, and high on neuroticism. However, since both studies eventually included only six individuals that fulfilled a formal BPD diagnosis, these findings are inconclusive for the clinical BPD population.

Building on these studies, our group has recently published a study using the ‘Thin Slices’ paradigm [15], in which we presented short videos of 52 BPD and age- and gender-matched healthy control (HC) participants (‘targets’) to two groups of student raters *(N*_1_ = 92, *N*_2_ = 44). These raters evaluated targets on the dimensions trustworthiness, likeability, and cooperativeness [16]. In both samples, BPD targets were rated as significantly less trustworthy and less likeable, and in one sample also as less cooperative. Notably, these findings were present without raters knowing anything about the targets’ mental health status, and therefore the influence of top-down processes such as stereotypes about mental illness was excluded. Moreover, the effects were markedly larger when we presented videos without (vs. with) the audio trace. This led us to conclude that raters must have relied on visual cues to form their judgments to a substantial degree. Following from this, we developed the ensuing research question, which visual cues raters could have used to form their negative judgments about BPD targets. The current study addressed this research question, specifically focusing on observable behaviors as possible cues. We focused on specific behaviors since these could potentially be modified in a therapeutic context, aiming to counter negative first impressions.

### The present study

To select probable cues for the trustworthiness, likeability and cooperativeness judgments obtained in the previous study [16], we did an extensive literature search on previous Thin Slices studies. Our search revealed *affect expression* as a central cue that raters consistently used to form judgments on constructs that measure some form of cooperativeness. Multiple studies found that raters use the intensity and frequency of positive versus negative affect expression (especially overt smiling) as a cue for agreeableness and altruism [17–23], and also cooperative behavior in economic games [22]. Beyond this, the display of positive facial affect was found to be a cue for likeability [20, 24], and for trustworthiness ratings [24–28]. In addition to facial affect display, the level of *eye contact* or looking straight into the camera emerged as another cue from the literature. In previous studies, the degree of eye contact was positively associated with agreeableness ratings [17, 29] as well as likeability [30–32] and trustworthiness ratings [33].

Based on this research, we selected positive affect (PA) display, negative affect (NA) display, and eye contact (EC) as potential cues for trustworthiness, likeability, and cooperativeness. Specifically, we expected these cues to mediate the observed relationship between BPD diagnostic status and poorer overall evaluation of trustworthiness, likeability and cooperativeness. Our assumption was also based on previous evidence that BPD individuals show low levels of PA display in experimental contexts, such as in reaction to PA induction [34], during a cyberball game [35], or in response to emotional pictures [36]. Likewise, BPD individuals displayed more NA (measured using facial electromyography) in previous studies when viewing emotional pictures [36–38] and during a problem-focused interview [39]. Previous evidence on reduced EC in BPD is lacking and therefore the examination of this cue is to be considered somewhat exploratory.

In sum, previous studies have provided first evidence that BPD individuals are evaluated negatively at a zero-acquaintance level, but it is yet unknown what these negative evaluations are based on. We identified PA and NA display as well as EC as potential cues for negative evaluations and expected BPD individuals to display more NA, less PA, and less EC. We expected that these observable cues would mediate the association between BPD status and negative evaluations on the traits trustworthiness, likeability, and cooperativeness.

## Methods

### Participants

A total of 101 participants were recruited via a participant pool of the University of Mannheim. Participants were between 18 and 55 years old (*M* = 23.7, *SD* = 4.5) and the majority of participants were female (60.4%), held a university entrance level degree (98.0%), and were students (84.2%) with a monthly income of less than 1000 Euros (76.2%).

### Procedure

Ethics approval for this study was granted by the Medical Ethics Committee II of the medical faculty Mannheim at Heidelberg University (protocol no. 2013-654 N-MA). At the beginning of the study, participants received detailed information about the study procedure and instructions. All participants gave written informed consent prior to participation. After providing demographic information, participants saw the 52 target videos and rated several nonverbal cues after each video. Participants enrolled in psychology majors received course credit for their participation.

### Material

#### Video-material

Detailed information on the generation of the video material is presented in Hepp et al. [16]. The video material comprised videos of 26 BPD and 26 age- and gender-matched HC participants. Participants were diagnosed by experienced clinicians using the structured clinical interview for DSM-IV SCID-I [40] and the international personality disorder examination IPDE [41]. Inclusion criteria were a current DSM-IV BPD diagnosis for the BPD group and the absence of any current or lifetime mental disorder or personality disorder for the HC group. In both groups, 46% of participants were men, and age did not differ significantly between the groups (*M*_BPD_ = 32.2, *SD*_BPD_ = 7.7; *M*_HC_ = 31.9, *SD*_HC_ = 8.0; *t*(50) = − 0.11, *p* = .916). Further demographic and diagnostic information on the target sample is presented in Hepp et al. [16].

Target participants were filmed while talking about their personal preferences (their favourite book, colour, movie, car, animal, food, hobby, and holiday destination). For the current study, videos were cut at 30 s and presented without the audio trace to exclude potential effects of speech content or prosody. Before the video-recording, target participants played an economic game called *dictator game* [42]. Participants were given an envelope containing 5 Euros in 50 cent coins and instructed to divide this money between themselves and an ‘unknown third person’. Participants extracted the money under complete anonymity and the recipients of the money remained unknown and had no way to react to the allocation. The amount of money shared in the dictator game is typically seen as an indicator of active cooperation or altruism. BPD and HC targets did not differ in the amount of money they actually shared, *t*(50) = 0.35, *p* = .727, *d* = 0.10.

#### Trustworthiness, likeability, and cooperativeness ratings

Trustworthiness, likeability, and cooperativeness (estimated money shared in dictator game) ratings for each target were obtained in a sample of 44 rater participants, which has previously been reported in Hepp et al. [16]. For the current study, we used the average ratings for each category per target. Ratings in all three categories were lower for BPD than for HC targets. BPD targets were evaluated as less trustworthy (*M*_BPD_ = 2.32, *SD*_BPD_ = 0.48, *M*_HC_ = 2.93, *SD*_HC_ = 0.51, *t*(50) = − 4.44, *p* < .001, *d* = 1.23), less likeable (*M*_BPD_ = 2.16, *SD*_BPD_ = 0.60, *M*_HC_ = 2.78, *SD*_HC_ = 0.71, *t*(50) = − 3.40, *p* = .001, *d* = 0.94), and less cooperative in terms of estimated money allocation in the dictator game (*M*_BPD_ = 2.02, *SD*_BPD_ = 0.27, *M*_HC_ = 2.27, *SD*_HC_ = 0.31, *t*(50) = − 3.17, *p* = .003, *d* = 0.88).

#### Cue ratings

Participants in the present study saw all 52 target videos and rated targets on nine different cues on a scale from 0 (“not at all”) to 5 (“very much”). The selection of cues included the three cues of interest for the current study: PA display (“The person often showed positive emotions, e.g. smiled.”), NA display (“The person often showed negative emotions, e.g. frowned”), and EC (“The person often looked directly into the camera”). In addition to these cues, we collected data on six further cues including “The person seemed distant and cool”, “The person seemed relaxed and confident”, “The person seemed educated and cultivated”, “The person is attractive”, “The person had a feminine appearance”, and “The person had a masculine appearance”. These variables were not related to the purpose of the current study, because they are not descriptors of a specific behaviour of the target, but rather pertain to global impressions. They are, thus, at a different level of granularity than the observable behaviours PA, NA and EC. With the purpose in mind of identifying directly modifiable behaviours, we have only analysed cues at the highest level of granularity and excluded variables that represented more global impressions. Though not analysed herein, we provide data for all additional cues in the online supplemental materials.

### Data analysis

To assess whether our previous finding that BPD individuals are seen as less trustworthy, less likeable, and less cooperative at zero acquaintance could be explained by PA, NA, and EC, we averaged each cue rating per target video and employed three mediation models using path analyses. We specified PA, NA, and EC (jointly) as mediators of the association between target group (BPD vs. HC) and trustworthiness, likeability, and cooperativeness ratings. Analyses were conducted in R, using the *sem* function of the *lavaan* package [43] with conventional standard errors. For the indirect effects, we additionally computed bootstrapped bias-corrected confidence intervals (*CI*_boot_) using the adjusted bootstrap percentile method.

## Results

**Fig. 1:**
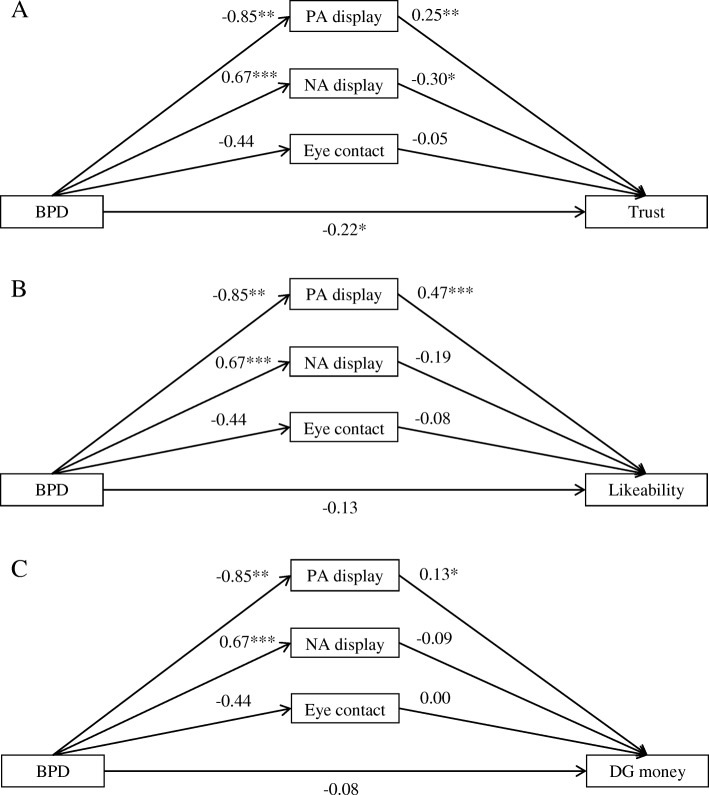
Unstandardized estimates of the path analyses that link BPD group to trustworthiness (panel **a**), likeability (panel **b**), and estimated amount of money shared in the dictator game (panel **c**) through the three mediating variables NA display, PA display, and eye contact (including a direct effect of group). Figure note*.* BPD = Borderline Personality Disorder, NA = negative affect, PA = positive affect. Significance indicated as * *p* < .05, ** *p* < .01, *** *p* < .001

Descriptive statistics for the cue ratings by target group are presented in Table 1. As expected, BPD targets were rated as showing less PA, and more NA than HC targets. However, target groups did not differ significantly in perceived EC (although the Bayes factor for this test was inconclusive). Results of the mediation models presented in Fig. 1 corroborate this picture, showing that group significantly predicted PA and NA, but not EC. Moreover, NA significantly predicted only likeability ratings, whereas PA predicted all three constructs, trustworthiness, likeability and cooperativeness. EC was not a significant predictor of any of the three criteria. In the context of the three mediators, group had a significant (negative) direct effect on trustworthiness, but not on likeability or cooperativeness. In other words, whether a target participant was in the BPD or the HC group significantly predicted how trustworthy, but not how likeable and cooperative they were rated, when PA, NA, and EC were statistically adjusted for.

**Table 1:** Descriptive statistics by target group for the cues positive affect (PA) display, negative affect (NA) display, and eye contact (EC)

	BPD		HC					
Cue	M	SD	M	SD	t(50)	p	d	BF_10_
PA	1.65	0.80	2.49	1.17	−3.04	.004	0.84	10.55
NA	1.69	0.63	1.02	0.59	3.98	<.001	1.10	109.98
EC	2.54	0.81	2.97	0.95	−1.79	.080	0.50	1.02

*Note*. BPD = targets with Borderline Personality Disorder, HC = healthy control targets

Results of between group t-tests are reported including two-sided Jeffrey-Zellner-Siow Bayes factors for evidence of H1 over H0 (BF_10_, H0: difference between groups = 0)

Indirect effects indicated that the group-trustworthiness association was significantly mediated by PA (*b* = − 0.22, *CI*_boot_ = [− 0.52; − 0.04], *p* = .037) and NA display (*b* = − 0.20, *CI*_boot_ = [− 0.47; − 0.03], *p* = .040) but not EC (*b* = 0.02, *CI*_boot_ = [− 0.03; 0.16], *p* = .490). The total indirect effect for the multiple mediation was significant (*b* = − 0.39, *CI*_boot_ = [− 0.65; − 0.19], *p* = .001).

The group-likeability association was significantly mediated only by PA display (*b* = − 0.40, *CI*_boot_ = [− 0.82; − 0.13], *p* = .013), but not NA (*b* = − 0.13, *CI*_boot_ = [− 0.40; 0.11], *p* = .247) or EC (*b* = 0.03, *CI*_boot_ = [− 0.04; 0.21], *p* = .428). The total indirect effect for the multiple mediation was significant (*b* = − 0.49, *CI*_boot_ = [− 0.83; − 0.21], *p* = .002).

The association between target group and estimated cooperativeness (i.e. money allocation in the dictator game) was not significantly mediated by either cue individually (PA: *b* = − 0.11, *CI*_boot_ = [− 0.28; 0.00], *p* = .094; NA: *b* = − 0.06, *CI*_boot_ = [− 0.23; 0.07], *p* = .345; EC: *b* = 0.00, *CI*_boot_ = [− 0.05; 0.06], *p* = .950) but the total indirect effect indicated a significant overall mediation (*b* = − 0.17, *CI*_boot_ = [− 0.33; − 0.06], *p* = .004).

## Discussion

The present study aimed to address the question why individuals with BPD are evaluated more negatively than healthy individuals at zero-acquaintance [12–14, 16]. We hypothesized that negative zero-acquaintance evaluations would be influenced by non-verbal cue displays by targets. To address this question, we used a target dataset with videos of 26 BPD and 26 age- and gender-matched HC individuals. In a previous study using the same target set, we observed that BPD targets were evaluated as less trustworthy, likeable, and cooperative [16]. Based on an extensive literature search, we identified PA display, NA display, and EC as likely nonverbal cues for negative ratings on the dimensions trustworthiness, likeability and cooperativeness. In the present study, we collected ratings of PA, NA, and EC for the 52 target videos from 101 raters. Next, we specified these ratings as mediators of the association between target group (BPD vs. HC) and trustworthiness, likeability as well as cooperativeness.

We found that BPD targets were rated as showing less PA and more NA than HC targets, which corroborates a number of previous findings [12, 34–39]. Importantly, PA display mediated the association between BPD group membership and likeability as well as between BPD group and trustworthiness. In contrast, NA display only mediated the association between target group and trustworthiness. Contrary to our hypothesis, the amount of EC did not significantly differ between the target groups and consequently did not mediate any of these associations. Moreover, neither cue individually mediated the group-cooperativeness association.

The importance of PA display for creating positive impressions on a range of global traits has previously been discussed (e.g., [25]), in the sense that PA signals overall approachability and encourages social bonds. Relating this back to the BPD population, and especially to interpersonal problems in BPD, it seems likely that low rates of PA display and associated poorer first impressions contribute to social isolation and interpersonal problems, because it might influence the initial behavior of an interaction partner. In other words, it seems likely that interpersonal problems in BPD are not only a result of impairments on the side of the BPD individual, but also of negative first impressions that other people form about those with BPD.

### Limitations and implications

A central limitation of the cue based design we employed herein is that it relies on subjective *ratings* of cues (even though this is a common practice with Thin Slices studies, e.g. 17). The sample we report herein *rated* BPD targets as showing less PA and more NA than HC targets, but this does not constitute a definite, objective measure of their affect display. For this reason, more recent approaches to cue based designs aim towards more objective measures of cues, for instance by using electromyography, or software that detects facial affect display based on patterns of muscle activation in the face^1^.

Additionally, the set of cues we chose, while strongly informed by previous evidence, is clearly not exhaustive and there are a multitude of other non-verbal behaviors raters could have relied on. Therefore, future studies are needed to replicate the present findings and extend the set of cues. Moreover, mediation analysis as used herein does not imply that significant mediators are at all causal or that another mediation model would not better explain the associations between group and trustworthiness/likeability/cooperativeness (see [44]). This again stresses the need for increasing objectivity and reliability of the cue ratings and extending the set of cues, or ideally even manipulating cues in experimental designs.

Nonetheless, affect expression provides a first, very clear target to address in further studies, and possibly also in therapeutic contexts. Therefore, it would be desirable to further refine the knowledge about how exactly affect expression informs global evaluations. Herein, we asked participants how ‘often’ targets showed behaviors such as smiling or frowning, thus assessing most likely the frequency of a certain display of affect. Other studies could distinguish between frequency, intensity, and duration, as these might have differential effects. For example, it would be helpful to know whether a brief but very intense smile could ‘compensate’ the influence of multiple minor frowns on the global expression.

In addition to further researching different types of cues and their relative relevance, future studies should address the question of diagnostic specificity. In the present study, we included only one clinical group of BPD individuals and thus cannot conclude whether the findings we made are an effect of specific BPD pathology, or psychopathology in general. Moreover, the target sample we report suffered from substantial comorbidity, which is common in BPD samples (e.g., [2]), but also entails the limitation that major comorbid conditions such as depression could have contributed to the observed lower rate of PA and more NA expression. Therefore, at this point, it would be premature to argue that our findings are specific to BPD. However, although few social bonds and interpersonal problems afflict patients of various disorders, it seems reasonable to study BPD individuals, as they are very prominently affected. Eventually, the finding we report herein may actually apply to other types of psychopathology as well and addressing affect display may be helpful for a range of patient groups.

The topic of affect display could be integrated in existing treatments of BPD such as dialectical behavior therapy [45], either by way of including it in skills training, or in other forms of social competence training. In any scenario, the inclusion of video-feedback could be particularly beneficial. The current results suggest a focus on PA display, as it had the strongest effects on global attributions of trustworthiness and likeability. Suppression of NA display, in contrast, may be a two-edged sword. On the one hand, the present findings and conceptualizations of NA as a withdrawal or threat signal would suggest including reduction of NA expression in therapeutic approaches. On the other hand, suppression of NA is (even outside of clinical samples) largely considered to be a poorly effective emotion regulation strategy that can actually increase subjective and physiological arousal (e.g., [46]). Therefore, therapeutic interventions to increase PA display seem the most likely starting point based on the limited evidence that is currently available.

## Conclusion

The present findings provide first evidence that a lack of facial PA expression and, to a lesser degree, also a high level of facial NA expression could contribute to negative evaluations of BPD individuals at zero-acquaintance. Thus, fostering BPD patients’ PA expression in therapeutic settings may help them elicit a more positive first impression in others. In our opinion, replication of the present findings and extension to a broader set of cues pertaining to non-verbal behaviors is the necessary next step. Any attempts to modify impression management in therapeutic settings can only come after this. Nonetheless, we hope that the potential benefit for patient populations will encourage other researchers to follow up on this question. If it were possible to teach patients to improve their impression management by way of learning to express PA more readily, and thus elicit a more positive first impression, this could increase BPD individuals’ opportunities to form social bonds and improve their chances at maintaining them.

## Abbreviations

BF_10_: Jeffrey-Zellner-Siow Bayes factor
BPD: Borderline Personality Disorder
CI_boot_: Bootstrapped bias-corrected confidence interval
DSM-IV: Diagnostic and statistical manual of mental disorders
EC: Eye contact
HC: Healthy control participants
IPDE: International personality disorder examination
NA: Negative affect
PA: Positive affect
SCID-I: Structured clinical interview for DSM-IV

## Footnotes

1 Since we wanted target participants to provide a video Thin Slice that was as naturalistic as possible, we chose not to attach electrodes to their faces or instruct them to restrict their movement, which would have been necessary to obtain reliable scores of facial affect based on computer algorithms.
